# 3,3′-{1,1′-Methyl­enebis[naphthalene-2,1-diylbis(oxymethyl­ene)]}dibenzonitrile

**DOI:** 10.1107/S1600536808009793

**Published:** 2008-05-03

**Authors:** Jie Xiao, Hong Zhao

**Affiliations:** a Ordered Matter Science Research Center, College of Chemistry and Chemical Engineering, Southeast University, Nanjing 210096, People’s Republic of China

## Abstract

The title compound, C_37_H_26_N_2_O_2_, was synthesized from 1,1′-methyl­enedinaphthalen-2-ol and 3-(bromo­methyl)­benzo­nitrile. The two naphthyl systems are almost perpendicular to each other [dihedral angle 83.3 (9)°] and the two cyano­benz­yloxy rings approximately parallel to each other [dihedral angle 15.5 (2)°].

## Related literature

For the application of nitrile derivatives in the synthesis of some heterocyclic mol­ecules, see: Radl *et al.* (2000 ▶). Fu & Zhao (2007 ▶) have reported benzonitrile compounds related to the title compound.
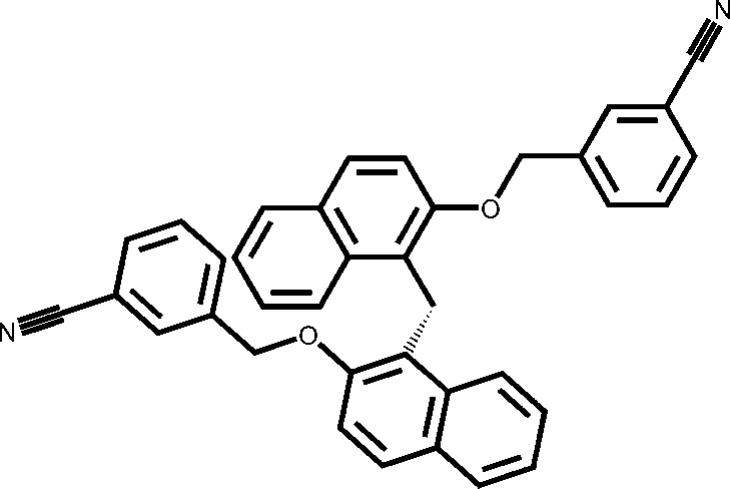


## Experimental

### 

#### Crystal data

C_37_H_26_N_2_O_2_
*M* *_r_* = 530.60
Triclinic, 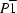

*a* = 9.3123 (19) Å
*b* = 12.130 (2) Å
*c* = 12.682 (3) Å
α = 79.71 (3)°
β = 86.58 (3)°
γ = 87.10 (3)°
*V* = 1405.9 (5) Å^3^
*Z* = 2
Mo *K*α radiation
μ = 0.08 mm^−1^
*T* = 293 (2) K
0.40 × 0.20 × 0.20 mm

#### Data collection

Rigaku Mercury2 diffractometer
Absorption correction: multi-scan (*CrystalClear*; Rigaku, 2005 ▶) *T*_min_ = 0.928, *T*_max_ = 0.976
13073 measured reflections
5505 independent reflections
2197 reflections with *I* > 2σ(*I*)
*R*_int_ = 0.074

#### Refinement

*R*[*F* ^2^ > 2σ(*F* ^2^)] = 0.069
*wR*(*F* ^2^) = 0.170
*S* = 0.92
5505 reflections
370 parameters
H-atom parameters constrained
Δρ_max_ = 0.18 e Å^−3^
Δρ_min_ = −0.18 e Å^−3^

### 

Data collection: *CrystalClear* (Rigaku, 2005 ▶); cell refinement: *CrystalClear*; data reduction: *CrystalClear*; program(s) used to solve structure: *SHELXTL/PC* (Sheldrick, 2008 ▶); program(s) used to refine structure: *SHELXTL/PC*; molecular graphics: *SHELXTL/PC*; software used to prepare material for publication: *SHELXTL/PC*.

## Supplementary Material

Crystal structure: contains datablocks I, global. DOI: 10.1107/S1600536808009793/zl2104sup1.cif

Structure factors: contains datablocks I. DOI: 10.1107/S1600536808009793/zl2104Isup2.hkl

Additional supplementary materials:  crystallographic information; 3D view; checkCIF report

## Figures and Tables

**Fig. 1.**
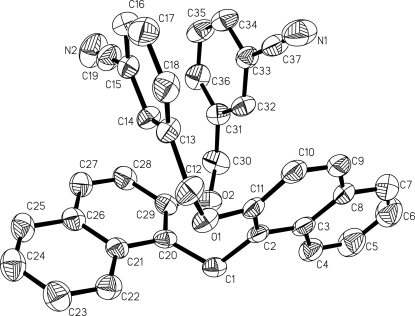
A view of the compound with the atomic numbering scheme. Displacement ellipsoids were drawn at the 30% probability level.

## supplementary crystallographic information

### Comment

Nitrile derivatives are important materials in the synthesis of some heterocyclic molecules (Radl *et al.*, 2000). Recently, we have reported a few benzonitrile compounds (Fu & Zhao, 2007). As an extension of our work on the structural characterization of nitrile compounds the structure of the title compound is reported here.

As shown in Fig. 1, the two naphthyl rings in the title compound are bridged by one C atom (C1) and are approximately perpendicular to each other with an dihedral angle of 83.3 (9)°. The two 3-cyanobenzyloxy rings, on the other hand are almost parallel to each other with a dihedral angle of 15.53 (0.23) °.

### Experimental

1,1'-Methylenedinaphthalen-2-ol (0.3 g, 1 mmol) and 3-(bromomethyl)benzonitrile (0.392 g, 2 mmol) were dissolved in acetone in the presence of K_2_CO_3_ (0.138 g, 1 mmol) and heated under reflux for 3 days. After the mixture was cooled to room temperature, the solution was filtered and the solvents removed in vacuum to afford a white precipitate of the title compound. Colourless crystals suitable for X-ray diffraction were obtained from a solution of 100 mg in 15 ml diethylether by slow evaporation after 3 days.

### Refinement

Positional parameters of all the H atoms were calculated geometrically and the H atoms were set to ride on the C and N atoms to which they are bonded, with *U*_iso_(H) = 1.2Ueq(C or N).

### Crystal data

**Table:**

C_37_H_26_N_2_O_2_	*Z* = 2
*M**_r_* = 530.60	*F*_000_ = 556.0
Triclinic, *P*1	*D*_x_ = 1.252 Mg m^−^^3^
Hall symbol: -P 1	Mo *K*α radiation λ = 0.71073 Å
*a* = 9.3123 (19) Å	Cell parameters from 7383 reflections
*b* = 12.130 (2) Å	θ = 3.2–27.5º
*c* = 12.682 (3) Å	µ = 0.08 mm^−^^1^
α = 79.71 (3)º	*T* = 293 (2) K
β = 86.58 (3)º	Block, colourless
γ = 87.10 (3)º	0.40 × 0.20 × 0.20 mm
*V* = 1405.9 (5) Å^3^	

### Data collection

**Table:**

Rigaku Mercury2 diffractometer	5505 independent reflections
Radiation source: fine-focus sealed tube	2197 reflections with *I* > 2σ(*I*)
Monochromator: graphite	*R*_int_ = 0.074
Detector resolution: 13.6612 pixels mm^-1^	θ_max_ = 26.0º
*T* = 293(2) K	θ_min_ = 3.2º
CCD_Profile_fitting scans	*h* = −11→11
Absorption correction: multi-scan(CrystalClear; Rigaku, 2005)	*k* = −14→14
*T*_min_ = 0.929, *T*_max_ = 0.976	*l* = −15→15
13073 measured reflections	

### Refinement

**Table:**

Refinement on *F*^2^	Secondary atom site location: difference Fourier map
Least-squares matrix: full	Hydrogen site location: inferred from neighbouring sites
*R*[*F*^2^ > 2σ(*F*^2^)] = 0.069	H-atom parameters constrained
*wR*(*F*^2^) = 0.170	*w* = 1/[σ^2^(*F*_o_^2^) + (0.0621*P*)^2^] where *P* = (*F*_o_^2^ + 2*F*_c_^2^)/3
*S* = 0.92	(Δ/σ)_max_ = 0.001
5505 reflections	Δρ_max_ = 0.18 e Å^−^^3^
370 parameters	Δρ_min_ = −0.18 e Å^−^^3^
Primary atom site location: structure-invariant direct methods	Extinction correction: none

### Special details

**Table:**

Geometry. All e.s.d.'s (except the e.s.d. in the dihedral angle between two l.s. planes) are estimated using the full covariance matrix. The cell e.s.d.'s are taken into account individually in the estimation of e.s.d.'s in distances, angles and torsion angles; correlations between e.s.d.'s in cell parameters are only used when they are defined by crystal symmetry. An approximate (isotropic) treatment of cell e.s.d.'s is used for estimating e.s.d.'s involving l.s. planes.
Refinement. Refinement of *F*^2^ against ALL reflections. The weighted *R*-factor *wR* and goodness of fit *S* are based on *F*^2^, conventional *R*-factors *R* are based on *F*, with *F* set to zero for negative *F*^2^. The threshold expression of *F*^2^ > σ(*F*^2^) is used only for calculating *R*-factors(gt) *etc*. and is not relevant to the choice of reflections for refinement. *R*-factors based on *F*^2^ are statistically about twice as large as those based on *F*, and *R*-factors based on ALL data will be even larger.

### Fractional atomic coordinates and isotropic or equivalent isotropic displacement parameters (Å^2^)

**Table:**

	*x*	*y*	*z*	*U*_iso_*/*U*_eq_	
C1	0.3027 (3)	0.9398 (2)	0.0249 (2)	0.0608 (9)	
H1A	0.2769	1.0188	0.0042	0.073*	
H1B	0.3652	0.9178	−0.0325	0.073*	
C2	0.1670 (3)	0.8741 (2)	0.0352 (2)	0.0523 (8)	
C3	0.1464 (4)	0.7897 (3)	−0.0270 (2)	0.0607 (9)	
C4	0.2492 (4)	0.7613 (3)	−0.1054 (3)	0.0763 (10)	
H4	0.3356	0.7980	−0.1171	0.092*	
C5	0.2242 (6)	0.6817 (4)	−0.1637 (3)	0.1065 (14)	
H5	0.2935	0.6650	−0.2151	0.128*	
C6	0.0968 (7)	0.6243 (4)	−0.1482 (4)	0.1182 (17)	
H6	0.0820	0.5695	−0.1887	0.142*	
C7	−0.0061 (5)	0.6482 (3)	−0.0739 (3)	0.0988 (13)	
H7	−0.0906	0.6091	−0.0637	0.119*	
C8	0.0143 (4)	0.7320 (3)	−0.0121 (3)	0.0692 (9)	
C9	−0.0890 (4)	0.7570 (3)	0.0656 (3)	0.0763 (10)	
H9	−0.1728	0.7170	0.0774	0.092*	
C10	−0.0707 (3)	0.8378 (3)	0.1242 (2)	0.0689 (9)	
H10	−0.1411	0.8540	0.1750	0.083*	
C11	0.0578 (3)	0.8971 (3)	0.1065 (2)	0.0537 (8)	
C12	−0.0149 (3)	1.0114 (3)	0.2408 (2)	0.0720 (10)	
H12A	−0.1122	1.0119	0.2173	0.086*	
H12B	0.0025	1.0864	0.2523	0.086*	
C13	−0.0040 (3)	0.9310 (2)	0.3465 (2)	0.0552 (8)	
C14	0.1197 (3)	0.8661 (2)	0.3723 (2)	0.0545 (8)	
H14	0.1972	0.8681	0.3224	0.065*	
C15	0.1290 (4)	0.7982 (2)	0.4718 (2)	0.0585 (8)	
C16	0.0151 (4)	0.7942 (3)	0.5460 (3)	0.0792 (11)	
H16	0.0210	0.7474	0.6124	0.095*	
C17	−0.1075 (4)	0.8592 (4)	0.5223 (3)	0.0910 (12)	
H17	−0.1842	0.8577	0.5729	0.109*	
C18	−0.1164 (3)	0.9276 (3)	0.4220 (3)	0.0742 (10)	
H18	−0.1996	0.9715	0.4060	0.089*	
C19	0.2585 (5)	0.7344 (3)	0.5006 (3)	0.0772 (10)	
C20	0.3869 (3)	0.9240 (2)	0.1264 (2)	0.0533 (8)	
C21	0.4046 (3)	1.0118 (3)	0.1852 (2)	0.0561 (8)	
C22	0.3464 (3)	1.1227 (3)	0.1556 (3)	0.0714 (9)	
H22	0.2943	1.1404	0.0940	0.086*	
C23	0.3649 (4)	1.2042 (3)	0.2149 (3)	0.0846 (11)	
H23	0.3250	1.2760	0.1935	0.102*	
C24	0.4431 (4)	1.1805 (3)	0.3075 (3)	0.0841 (11)	
H24	0.4561	1.2365	0.3470	0.101*	
C25	0.4999 (3)	1.0755 (3)	0.3396 (3)	0.0777 (10)	
H25	0.5511	1.0600	0.4017	0.093*	
C26	0.4827 (3)	0.9889 (3)	0.2803 (3)	0.0624 (9)	
C27	0.5416 (4)	0.8801 (3)	0.3132 (3)	0.0774 (10)	
H27	0.5917	0.8647	0.3758	0.093*	
C28	0.5275 (3)	0.7970 (3)	0.2567 (3)	0.0739 (10)	
H28	0.5684	0.7259	0.2797	0.089*	
C29	0.4503 (3)	0.8193 (3)	0.1627 (3)	0.0596 (8)	
C30	0.4664 (4)	0.6241 (3)	0.1401 (3)	0.0855 (11)	
H30A	0.5641	0.6153	0.1636	0.103*	
H30B	0.4609	0.5814	0.0828	0.103*	
C31	0.3651 (4)	0.5757 (3)	0.2329 (3)	0.0658 (9)	
C32	0.2190 (4)	0.6039 (3)	0.2314 (2)	0.0680 (9)	
H32	0.1828	0.6548	0.1745	0.082*	
C33	0.1263 (4)	0.5558 (3)	0.3156 (3)	0.0626 (8)	
C34	0.1783 (4)	0.4822 (3)	0.4006 (3)	0.0774 (10)	
H34	0.1158	0.4516	0.4570	0.093*	
C35	0.3224 (5)	0.4532 (3)	0.4027 (3)	0.0867 (12)	
H35	0.3577	0.4018	0.4597	0.104*	
C36	0.4153 (4)	0.5011 (3)	0.3195 (3)	0.0812 (11)	
H36	0.5133	0.4826	0.3221	0.097*	
C37	−0.0261 (5)	0.5865 (3)	0.3164 (3)	0.0762 (10)	
N1	−0.1484 (4)	0.6073 (3)	0.3198 (3)	0.1080 (12)	
N2	0.3622 (4)	0.6844 (3)	0.5255 (3)	0.1119 (12)	
O1	0.0827 (2)	0.98473 (17)	0.15795 (15)	0.0632 (6)	
O2	0.4363 (2)	0.74029 (18)	0.09827 (17)	0.0715 (6)	

### Atomic displacement parameters (Å^2^)

**Table:**

	*U*^11^	*U*^22^	*U*^33^	*U*^12^	*U*^13^	*U*^23^
C1	0.063 (2)	0.063 (2)	0.0519 (19)	−0.0079 (17)	0.0066 (17)	0.0001 (15)
C2	0.058 (2)	0.061 (2)	0.0338 (16)	−0.0052 (16)	−0.0026 (15)	0.0048 (14)
C3	0.077 (2)	0.060 (2)	0.0403 (18)	0.0011 (19)	−0.0095 (18)	0.0054 (16)
C4	0.102 (3)	0.074 (2)	0.054 (2)	−0.001 (2)	−0.005 (2)	−0.0130 (19)
C5	0.140 (4)	0.106 (4)	0.078 (3)	0.009 (3)	−0.009 (3)	−0.030 (3)
C6	0.169 (5)	0.089 (3)	0.103 (4)	−0.009 (4)	−0.040 (4)	−0.024 (3)
C7	0.113 (4)	0.096 (3)	0.088 (3)	−0.026 (3)	−0.031 (3)	−0.005 (3)
C8	0.083 (3)	0.069 (2)	0.053 (2)	−0.011 (2)	−0.023 (2)	0.0061 (18)
C9	0.069 (2)	0.088 (3)	0.065 (2)	−0.018 (2)	−0.020 (2)	0.016 (2)
C10	0.055 (2)	0.093 (3)	0.051 (2)	−0.002 (2)	−0.0088 (17)	0.0085 (19)
C11	0.058 (2)	0.061 (2)	0.0388 (17)	0.0039 (17)	−0.0137 (16)	0.0025 (15)
C12	0.063 (2)	0.091 (3)	0.060 (2)	0.0176 (19)	−0.0102 (19)	−0.0102 (19)
C13	0.0423 (18)	0.075 (2)	0.0481 (18)	0.0035 (16)	−0.0009 (15)	−0.0132 (16)
C14	0.0498 (19)	0.066 (2)	0.0461 (18)	0.0036 (16)	−0.0017 (15)	−0.0074 (15)
C15	0.062 (2)	0.062 (2)	0.0524 (19)	−0.0055 (17)	−0.0116 (18)	−0.0072 (17)
C16	0.093 (3)	0.089 (3)	0.053 (2)	−0.024 (2)	−0.004 (2)	−0.0009 (19)
C17	0.068 (3)	0.133 (4)	0.072 (3)	−0.023 (3)	0.017 (2)	−0.020 (2)
C18	0.047 (2)	0.104 (3)	0.075 (2)	−0.0034 (19)	−0.0025 (19)	−0.024 (2)
C19	0.096 (3)	0.071 (2)	0.064 (2)	0.008 (2)	−0.024 (2)	−0.0060 (18)
C20	0.0409 (18)	0.059 (2)	0.0536 (19)	−0.0040 (16)	0.0058 (15)	0.0047 (16)
C21	0.0402 (18)	0.065 (2)	0.060 (2)	−0.0108 (16)	0.0035 (16)	−0.0036 (17)
C22	0.062 (2)	0.075 (2)	0.074 (2)	−0.0013 (19)	−0.0087 (19)	−0.003 (2)
C23	0.081 (3)	0.075 (3)	0.100 (3)	0.000 (2)	−0.007 (2)	−0.022 (2)
C24	0.064 (3)	0.102 (3)	0.094 (3)	−0.009 (2)	−0.001 (2)	−0.038 (2)
C25	0.051 (2)	0.103 (3)	0.084 (3)	−0.015 (2)	−0.0081 (19)	−0.025 (2)
C26	0.0375 (18)	0.075 (2)	0.073 (2)	−0.0094 (17)	−0.0015 (17)	−0.0056 (19)
C27	0.056 (2)	0.092 (3)	0.083 (3)	−0.013 (2)	−0.0246 (19)	−0.002 (2)
C28	0.048 (2)	0.073 (2)	0.097 (3)	−0.0018 (18)	−0.021 (2)	0.002 (2)
C29	0.0405 (18)	0.066 (2)	0.071 (2)	−0.0077 (17)	0.0068 (17)	−0.0100 (19)
C30	0.077 (3)	0.070 (3)	0.105 (3)	0.011 (2)	0.007 (2)	−0.011 (2)
C31	0.068 (2)	0.056 (2)	0.070 (2)	0.0080 (18)	0.0004 (19)	−0.0091 (17)
C32	0.077 (3)	0.067 (2)	0.054 (2)	0.0040 (19)	−0.0086 (19)	0.0052 (16)
C33	0.072 (2)	0.057 (2)	0.058 (2)	0.0037 (18)	−0.0107 (19)	−0.0052 (17)
C34	0.101 (3)	0.066 (2)	0.059 (2)	0.011 (2)	0.002 (2)	−0.0009 (18)
C35	0.122 (4)	0.065 (2)	0.066 (3)	0.021 (2)	−0.007 (3)	0.0030 (19)
C36	0.089 (3)	0.060 (2)	0.091 (3)	0.027 (2)	−0.024 (2)	−0.007 (2)
C37	0.087 (3)	0.084 (3)	0.051 (2)	−0.002 (2)	−0.002 (2)	0.0059 (17)
N1	0.078 (2)	0.142 (3)	0.088 (2)	0.003 (2)	0.005 (2)	0.018 (2)
N2	0.131 (3)	0.099 (3)	0.106 (3)	0.038 (2)	−0.054 (2)	−0.015 (2)
O1	0.0631 (14)	0.0748 (15)	0.0488 (12)	0.0082 (12)	−0.0002 (11)	−0.0070 (11)
O2	0.0707 (15)	0.0648 (15)	0.0739 (15)	0.0050 (12)	0.0047 (12)	−0.0038 (12)

### Geometric parameters (Å, °)

**Table:**

C1—C2	1.515 (4)	C18—H18	0.9300
C1—C20	1.525 (4)	C19—N2	1.144 (4)
C1—H1A	0.9700	C20—C29	1.386 (4)
C1—H1B	0.9700	C20—C21	1.427 (4)
C2—C11	1.372 (4)	C21—C22	1.419 (4)
C2—C3	1.425 (4)	C21—C26	1.423 (4)
C3—C4	1.414 (4)	C22—C23	1.369 (4)
C3—C8	1.432 (4)	C22—H22	0.9300
C4—C5	1.354 (5)	C23—C24	1.397 (5)
C4—H4	0.9300	C23—H23	0.9300
C5—C6	1.392 (6)	C24—C25	1.357 (4)
C5—H5	0.9300	C24—H24	0.9300
C6—C7	1.360 (5)	C25—C26	1.417 (4)
C6—H6	0.9300	C25—H25	0.9300
C7—C8	1.417 (5)	C26—C27	1.405 (4)
C7—H7	0.9300	C27—C28	1.352 (4)
C8—C9	1.397 (4)	C27—H27	0.9300
C9—C10	1.355 (4)	C28—C29	1.407 (4)
C9—H9	0.9300	C28—H28	0.9300
C10—C11	1.413 (4)	C29—O2	1.381 (3)
C10—H10	0.9300	C30—O2	1.434 (3)
C11—O1	1.379 (3)	C30—C31	1.515 (4)
C12—O1	1.418 (3)	C30—H30A	0.9700
C12—C13	1.516 (4)	C30—H30B	0.9700
C12—H12A	0.9700	C31—C36	1.382 (4)
C12—H12B	0.9700	C31—C32	1.387 (4)
C13—C18	1.373 (4)	C32—C33	1.394 (4)
C13—C14	1.384 (4)	C32—H32	0.9300
C14—C15	1.382 (4)	C33—C34	1.367 (4)
C14—H14	0.9300	C33—C37	1.449 (5)
C15—C16	1.373 (4)	C34—C35	1.370 (5)
C15—C19	1.429 (5)	C34—H34	0.9300
C16—C17	1.371 (5)	C35—C36	1.386 (4)
C16—H16	0.9300	C35—H35	0.9300
C17—C18	1.394 (4)	C36—H36	0.9300
C17—H17	0.9300	C37—N1	1.154 (4)
			
C2—C1—C20	114.8 (2)	C17—C18—H18	119.5
C2—C1—H1A	108.6	N2—C19—C15	178.6 (4)
C20—C1—H1A	108.6	C29—C20—C21	118.5 (3)
C2—C1—H1B	108.6	C29—C20—C1	118.3 (3)
C20—C1—H1B	108.6	C21—C20—C1	123.2 (3)
H1A—C1—H1B	107.6	C22—C21—C26	116.8 (3)
C11—C2—C3	118.1 (3)	C22—C21—C20	123.8 (3)
C11—C2—C1	119.0 (3)	C26—C21—C20	119.4 (3)
C3—C2—C1	122.9 (3)	C23—C22—C21	121.8 (3)
C4—C3—C2	123.2 (3)	C23—C22—H22	119.1
C4—C3—C8	117.7 (3)	C21—C22—H22	119.1
C2—C3—C8	119.1 (3)	C22—C23—C24	120.6 (4)
C5—C4—C3	121.2 (4)	C22—C23—H23	119.7
C5—C4—H4	119.4	C24—C23—H23	119.7
C3—C4—H4	119.4	C25—C24—C23	119.8 (4)
C4—C5—C6	121.2 (4)	C25—C24—H24	120.1
C4—C5—H5	119.4	C23—C24—H24	120.1
C6—C5—H5	119.4	C24—C25—C26	121.2 (3)
C7—C6—C5	120.3 (4)	C24—C25—H25	119.4
C7—C6—H6	119.9	C26—C25—H25	119.4
C5—C6—H6	119.9	C27—C26—C25	121.4 (3)
C6—C7—C8	120.6 (4)	C27—C26—C21	118.9 (3)
C6—C7—H7	119.7	C25—C26—C21	119.8 (3)
C8—C7—H7	119.7	C28—C27—C26	122.0 (3)
C9—C8—C7	121.6 (4)	C28—C27—H27	119.0
C9—C8—C3	119.2 (3)	C26—C27—H27	119.0
C7—C8—C3	119.1 (4)	C27—C28—C29	119.3 (3)
C10—C9—C8	121.9 (4)	C27—C28—H28	120.3
C10—C9—H9	119.1	C29—C28—H28	120.3
C8—C9—H9	119.1	O2—C29—C20	115.3 (3)
C9—C10—C11	118.5 (3)	O2—C29—C28	122.8 (3)
C9—C10—H10	120.7	C20—C29—C28	121.9 (3)
C11—C10—H10	120.7	O2—C30—C31	114.0 (3)
C2—C11—O1	114.2 (3)	O2—C30—H30A	108.7
C2—C11—C10	123.0 (3)	C31—C30—H30A	108.7
O1—C11—C10	122.7 (3)	O2—C30—H30B	108.7
O1—C12—C13	114.0 (2)	C31—C30—H30B	108.7
O1—C12—H12A	108.7	H30A—C30—H30B	107.6
C13—C12—H12A	108.7	C36—C31—C32	118.5 (3)
O1—C12—H12B	108.7	C36—C31—C30	121.1 (3)
C13—C12—H12B	108.7	C32—C31—C30	120.4 (3)
H12A—C12—H12B	107.6	C31—C32—C33	119.8 (3)
C18—C13—C14	118.7 (3)	C31—C32—H32	120.1
C18—C13—C12	119.2 (3)	C33—C32—H32	120.1
C14—C13—C12	122.0 (3)	C34—C33—C32	120.7 (3)
C15—C14—C13	120.6 (3)	C34—C33—C37	118.9 (3)
C15—C14—H14	119.7	C32—C33—C37	120.4 (3)
C13—C14—H14	119.7	C33—C34—C35	120.1 (3)
C16—C15—C14	120.2 (3)	C33—C34—H34	120.0
C16—C15—C19	118.9 (3)	C35—C34—H34	120.0
C14—C15—C19	120.9 (3)	C34—C35—C36	119.6 (3)
C17—C16—C15	120.0 (3)	C34—C35—H35	120.2
C17—C16—H16	120.0	C36—C35—H35	120.2
C15—C16—H16	120.0	C31—C36—C35	121.3 (3)
C16—C17—C18	119.6 (3)	C31—C36—H36	119.3
C16—C17—H17	120.2	C35—C36—H36	119.3
C18—C17—H17	120.2	N1—C37—C33	177.5 (4)
C13—C18—C17	121.0 (3)	C11—O1—C12	120.4 (3)
C13—C18—H18	119.5	C29—O2—C30	119.5 (3)
			
C20—C1—C2—C11	62.9 (3)	C1—C20—C21—C22	−0.2 (4)
C20—C1—C2—C3	−117.8 (3)	C29—C20—C21—C26	−1.7 (4)
C11—C2—C3—C4	177.9 (3)	C1—C20—C21—C26	178.9 (2)
C1—C2—C3—C4	−1.4 (4)	C26—C21—C22—C23	0.3 (5)
C11—C2—C3—C8	−0.5 (4)	C20—C21—C22—C23	179.4 (3)
C1—C2—C3—C8	−179.8 (3)	C21—C22—C23—C24	0.3 (5)
C2—C3—C4—C5	−179.0 (3)	C22—C23—C24—C25	−0.7 (5)
C8—C3—C4—C5	−0.6 (5)	C23—C24—C25—C26	0.6 (5)
C3—C4—C5—C6	−0.4 (6)	C24—C25—C26—C27	179.9 (3)
C4—C5—C6—C7	0.5 (7)	C24—C25—C26—C21	0.0 (5)
C5—C6—C7—C8	0.5 (7)	C22—C21—C26—C27	179.6 (3)
C6—C7—C8—C9	−179.2 (4)	C20—C21—C26—C27	0.5 (4)
C6—C7—C8—C3	−1.5 (5)	C22—C21—C26—C25	−0.4 (4)
C4—C3—C8—C9	179.3 (3)	C20—C21—C26—C25	−179.6 (3)
C2—C3—C8—C9	−2.3 (4)	C25—C26—C27—C28	−179.2 (3)
C4—C3—C8—C7	1.5 (4)	C21—C26—C27—C28	0.7 (5)
C2—C3—C8—C7	180.0 (3)	C26—C27—C28—C29	−0.7 (5)
C7—C8—C9—C10	−179.4 (3)	C21—C20—C29—O2	−176.2 (2)
C3—C8—C9—C10	2.9 (5)	C1—C20—C29—O2	3.3 (4)
C8—C9—C10—C11	−0.8 (5)	C21—C20—C29—C28	1.7 (4)
C3—C2—C11—O1	−175.9 (2)	C1—C20—C29—C28	−178.8 (3)
C1—C2—C11—O1	3.5 (3)	C27—C28—C29—O2	177.3 (3)
C3—C2—C11—C10	2.8 (4)	C27—C28—C29—C20	−0.5 (5)
C1—C2—C11—C10	−177.9 (3)	O2—C30—C31—C36	−140.6 (3)
C9—C10—C11—C2	−2.2 (4)	O2—C30—C31—C32	40.7 (4)
C9—C10—C11—O1	176.4 (3)	C36—C31—C32—C33	−0.9 (5)
O1—C12—C13—C18	−161.5 (3)	C30—C31—C32—C33	177.8 (3)
O1—C12—C13—C14	23.0 (4)	C31—C32—C33—C34	0.9 (5)
C18—C13—C14—C15	0.8 (4)	C31—C32—C33—C37	178.6 (3)
C12—C13—C14—C15	176.4 (3)	C32—C33—C34—C35	−1.2 (5)
C13—C14—C15—C16	0.2 (5)	C37—C33—C34—C35	−178.9 (3)
C13—C14—C15—C19	−177.4 (3)	C33—C34—C35—C36	1.5 (5)
C14—C15—C16—C17	−1.1 (5)	C32—C31—C36—C35	1.2 (5)
C19—C15—C16—C17	176.5 (3)	C30—C31—C36—C35	−177.5 (3)
C15—C16—C17—C18	1.1 (5)	C34—C35—C36—C31	−1.5 (5)
C14—C13—C18—C17	−0.8 (5)	C2—C11—O1—C12	−174.4 (2)
C12—C13—C18—C17	−176.6 (3)	C10—C11—O1—C12	7.0 (4)
C16—C17—C18—C13	−0.1 (5)	C13—C12—O1—C11	75.5 (3)
C2—C1—C20—C29	66.3 (3)	C20—C29—O2—C30	−165.0 (2)
C2—C1—C20—C21	−114.2 (3)	C28—C29—O2—C30	17.0 (4)
C29—C20—C21—C22	179.2 (3)	C31—C30—O2—C29	64.3 (4)
